# Rapid PCR Testing Reduces Antibiotic Prescribing in Children Presenting to Hospital With Lower Respiratory Tract Infections due to Influenza

**DOI:** 10.1111/irv.13265

**Published:** 2024-02-29

**Authors:** Tony Walls, Emily Bowen, Ava Elsmore, Nathanael Lucas

**Affiliations:** ^1^ Department of Paediatrics University of Otago Christchurch UK; ^2^ Paediatric Department Te Whatu Ora (HealthNZ) Waitaha Canterbury UK

**Keywords:** children, influenza, PCR testing

Dear Sir/Madam

We have previously demonstrated that in young children with lower respiratory tract infections (LRTI) admitted to hospital, identifying a virus on multiplex PCR testing of nasopharyngeal swabs (NPS) can lead to a reduction in antibiotic usage [1]. Rapid availability of viral PCR test results appears to provide additional antimicrobial stewardship benefits and can further reduce unnecessary antibiotic prescribing.

Historically, Christchurch Hospital Paediatric Department in New Zealand has used a multiplex viral PCR testing at clinician discretion which would have an average turnaround time of 24 h (non‐rapid PCR). During the recent COVID‐19 pandemic, rapid PCR testing was introduced to routinely screen all children presenting with influenza‐like illness (ILI) for COVID‐19, influenza A or B and respiratory syncytial virus. The laboratories used a variety of platforms, all with a turnaround time of approximately 1 h (rapid PCR) to allow for appropriate identification of pathogens for infection control purposes and possible treatment.

We compared two cohorts of children with community‐acquired, influenza PCR‐positive LRTI from different winter seasons: (1) children who had non‐rapid multiplex PCR testing during the years 2012–2015 [1] and (2) children who tested positive with rapid PCR during 2022 [2]. The diagnosis of LRTI was made in each cohort following detailed review of inpatient notes. These studies were both carried out in a single centre with the same antibiotic guidelines in place over the different time periods. Our department has historically seldom prescribed oseltamivir, and its use was at the discretion of the admitting clinician.

In the non‐rapid PCR influenza cohort, 26 of 68 (38.2%) children with LRTI were treated with antibiotics in hospital. In comparison, the children in the rapid PCR influenza cohort with LRTI were significantly less likely to be prescribed antibiotics in hospital—40 children out of 306 (13.1%, *p* = <0.001). In this latter cohort, only nine children (3%) received oseltamivir, and there were no ICU admissions. All but one of the cases were due to influenza A.

Our clinical impression had been that having access to rapid PCR results makes it easier for resident medical officers to avoid prescribing antibiotics for young children with probable viral LRTI. These data for the children with proven influenza infection support the hypothesis that the introduction of rapid PCR testing has led to a reduction in unnecessary antibiotic prescribing.

There was a change in the use of viral testing between the different cohorts with an NPS being part of the initial assessment on admission of any child with ILI in 2022. This compares to the clinician‐directed testing in the earlier cohort, where testing was less frequent. While rapid testing appears to have influenced antibiotic prescribing, it is possible that other factors such as a greater proportion of less unwell children in our later cohort contributed to the reduction in the use of antibiotics.

The introduction of rapid PCR testing of children with ILI in hospital has had many benefits in the COVID‐19 era. It makes infection control precautions easier to manage and provides more information for families and clinicians alike. We have shown that it is also a valuable tool for antimicrobial stewardship, giving resident medical officers confidence not to prescribe antibiotics in children with influenza infection and reducing unnecessary antimicrobial prescribing.

## Author Contributions

**Tony Walls:** Conceptualization; data curation; writing—original draft; writing—review and editing. **Emily Bowen:** Conceptualization; data curation; writing—review and editing. **Ava Elsmore:** Data curation; formal analysis; writing—review and editing. **Nathanael Lucas:** Formal analysis; writing—original draft; writing—review and editing.

## Conflicts of Interest

The authors declare no conflicts of interest.

### Peer Review

The peer review history for this article is available at https://www.webofscience.com/api/gateway/wos/peer‐review/10.1111/irv.13265.

## References

[1] T. Walls , E. Stark , P. Pattemore , and L. Jennings , “Missed Opportunities for Antimicrobial Stewardship in Pre‐School Children Admitted to Hospital with Lower Respiratory Tract Infection,” JPCH 53, no. 6 (2017): 569–571, 10.1111/jpc.13506. PMC7166398 28333409

[2] A. Elsmore , M. Bowen , N. Lucas , and T. Walls , “Clinical Characteristics of Children <16 Years of Age Admitted to Christchurch Hospital with PCR‐Confirmed Influenza,” 2022. Poster presentation Te Niwha Infectious Diseases and Pandemic Preparedness Summit November 2023 Christchurch, New Zealand.

